# Nanofabrication Techniques for Enhancing Plant–Microbe Interactions in Sustainable Agriculture

**DOI:** 10.3390/nano15141086

**Published:** 2025-07-14

**Authors:** Wajid Zaman, Atif Ali Khan Khalil, Adnan Amin, Sajid Ali

**Affiliations:** 1 Department of Life Sciences, Yeungnam University, Gyeongsan 38541, Republic of Korea; wajidzaman@yu.ac.kr; 2 Department of Biotechnology, Yeungnam University, Gyeongsan 38541, Republic of Korea; atif.ali@yu.ac.kr; 3 Department of Horticulture and Life Science, Yeungnam University, Gyeongsan 38541, Republic of Korea

**Keywords:** nanomaterials, plant-microbe interactions, nanosensors, electrospinning, nanocoatings, microbial inoculants, surface properties, sustainable agriculture

## Abstract

Nanomaterials have emerged as a transformative technology in agricultural science, offering innovative solutions to improve plant–microbe interactions and crop productivity. The unique properties, such as high surface area, tunability, and reactivity, of nanomaterials, including nanoparticles, carbon-based materials, and electrospun fibers, render them ideal for applications such as nutrient delivery systems, microbial inoculants, and environmental monitoring. This review explores various types of nanomaterials employed in agriculture, focusing on their role in enhancing microbial colonization and soil health and optimizing plant growth. Key nanofabrication techniques, including top-down and bottom-up manufacturing, electrospinning, and nanoparticle synthesis, are discussed in relation to controlled release systems and microbial inoculants. Additionally, the influence of surface properties such as charge, porosity, and hydrophobicity on microbial adhesion and colonization is examined. Moreover, the potential of nanocoatings and electrospun fibers to enhance seed protection and promote beneficial microbial interactions is investigated. Furthermore, the integration of nanosensors for detecting pH, reactive oxygen species, and metabolites offers real-time insights into the biochemical dynamics of plant–microbe systems, applicable to precision farming. Finally, the environmental and safety considerations regarding the use of nanomaterials, including biodegradability, nanotoxicity, and regulatory concerns, are addressed. This review emphasizes the potential of nanomaterials to revolutionize sustainable agricultural practices by improving crop health, nutrient efficiency, and environmental resilience.

## 1. Introduction

Plant–microbe interactions influence the functioning of ecosystems, especially in agricultural systems, in which these interactions contribute significantly to plant growth, nutrient cycling, disease resistance, and stress tolerance [1]. Moreover, these interactions, occurring primarily in the rhizosphere, involve a diverse range of microorganisms such as bacteria, fungi, and archaea that form symbiotic relationships with plants [2]. For example, nitrogen-fixing rhizobia species form symbioses with leguminous plants, thereby enhancing nitrogen availability in soil, essential for plant growth [3]. Similarly, mycorrhizal fungi enhance phosphorus uptake, an essential nutrient for plant development, whereas plant growth-promoting rhizobacteria (PGPR) improve plant health by producing growth hormones and outcompeting pathogens [4]. These naturally occurring relationships are vital for maintaining soil health and reducing dependency on chemical fertilizers and pesticides, rendering these relationships integral to sustainable agriculture practices [5].

Traditional methods of studying and engineering plant–microbe systems have limitations [6]. Techniques such as culturing, in which individual microbial species are isolated and studied under artificial conditions, fail to capture the complex interactions occurring in natural environments [7]. This approach often overlooks the role of microbial communities, as well as their interspecies interactions and dynamic behaviors within the rhizosphere. Although genetic manipulation tools are powerful, they may not fully represent the complexity of microbial ecosystems, as these tools tend to focus on single species or genes, neglecting the broad network of interactions [8]. Additionally, the spatial distribution and temporal dynamics of these microorganisms in response to various environmental stimuli remain largely uncharacterized, owing to the challenges in the real-time monitoring of these interactions [9].

Nanofabrication approaches offer a promising means to address the limitations of conventional tools in studying and engineering plant–microbe interactions [10]. Nanotechnology enables the formation of highly controlled environments at the nanoscale, in which surfaces and materials can be precisely manipulated [11]. Researchers can design materials that mimic plant root surfaces or optimize microbial interactions at a molecular level using nanofabrication techniques [12]. For instance, nanostructured surfaces can be engineered to enhance microbial attachment or influence microbial behavior, increasing the efficiency of nutrient cycling and suppressing diseases. Moreover, nanoparticles can be used as carriers for microbial inoculants, ensuring stability and targeted delivery to plant roots, thereby improving the effectiveness of these inoculants in promoting plant growth and health [13]. These advancements present new opportunities for sustainably enhancing agricultural productivity by reducing the need for chemical inputs.

Furthermore, nanofabrication techniques enable the development of advanced delivery systems for biofertilizers, biopesticides, and plant growth regulators, allowing for the controlled and efficient release of these substances into the soil or directly onto plant roots [14]. By encapsulating these bioactive agents in nanoparticles, the release of these agents can be regulated over time, preventing leaching and ensuring that the plants receive a consistent supply of essential nutrients and protection against pathogens [15]. Such controlled release minimizes environmental impact by reducing the application frequency and the associated risks of chemical runoff. Additionally, the integration of microfluidic and biosensor technologies enables real-time monitoring of microbial colonization and root exudate profiles, clarifying the physiological responses of plants and microbes under different environmental conditions [16].

This review explores the various nanofabrication techniques employed to enhance plant–microbe interactions in sustainable agriculture. This review encompasses a wide range of nanofabrication methods, including top-down and bottom-up approaches, and the applications in fabricating engineered surfaces and nanoparticle-based delivery systems. Moreover, the use of nanoformulations for microbial inoculants, the role of nanostructured surfaces in improving root–microbe interactions, and the potential of applying nanotechnology in monitoring plant–microbe dynamics using advanced biosensors and microfluidics are highlighted. Additionally, the environmental and safety considerations associated with the use of nanomaterials in agriculture in terms of biodegradability, toxicity to soil microbiota and plants, and regulatory outlook are discussed. This review provides an overview of the current advancements in nanofabrication for plant–microbe interactions, clarifying the integration of nanotechnology into agricultural practices to promote sustainability and reduce dependency on traditional chemical inputs.

## 2. Overview of Nanofabrication Techniques

**Figure 1:**
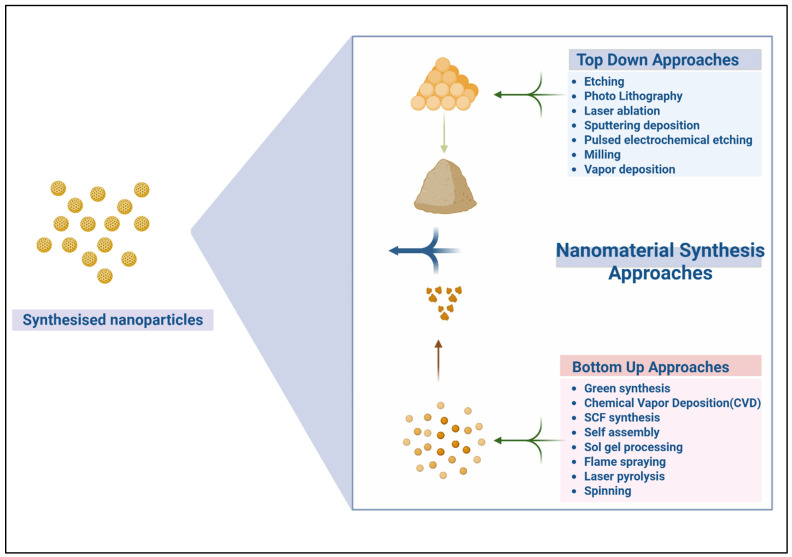
Various modern approaches for synthesizing nanoparticles.

Nanofabrication techniques have revolutionized the development of nanoscale materials, offering innovative means to manipulate and engineer materials and systems with high precision [17]. These techniques can be broadly classified into two categories, namely, top-down and bottom-up nanomanufacturing. Both approaches possess distinct advantages and applications (Figure 1). Additionally, the complementary nature of these approaches renders them crucial for advancing various fields, including agriculture [18]. The ability to control structures at the nanoscale allows researchers to fabricate highly functionalized surfaces, develop novel delivery systems, and design materials with unique properties that can enhance plant–microbe interactions [19]. Nanofabrication is essential for fabricating surfaces for microbe adhesion, nanostructured coatings for seed treatment, and nanoformulations for the controlled release of bioactive compounds, all of which contribute to sustainable agricultural practices [15].

### 2.1. Top-Down vs. Bottom-Up Nanomanufacturing

Top-down nanomanufacturing refers to the process of fabricating nanoscale structures by starting with bulk materials and systematically reducing the sizes of these materials. Photolithography, etching, and milling are some common top-down nanomanufacturing approaches. For example, photolithography involves the projection of light onto a material surface covered with a photosensitive film to fabricate intricate patterns [20]. These patterns can subsequently be transferred to the material, yielding precise nanoscale structures. This method is widely used in the semiconductor industry and applied in agricultural research for fabricating nanopatterned surfaces that promote microbial adhesion and growth. Etching uses reactive ions to remove material and generate nanoscale features; therefore, this technique is useful for fabricating nanoarrays or micropatterns on various substrates that can enhance plant–microbe interactions [21].

By contrast, bottom-up nanomanufacturing can be used to construct nanoscale materials from atomic or molecular units, building the structure layer-wise. Self-assembly, chemical vapor deposition (CVD), and sol–gel processing are some such approaches [18]. In self-assembly, molecules spontaneously organize themselves into specific structures based on their intrinsic properties; therefore, this approach is efficient and versatile for producing nanostructured materials. In contrast, CVD involves the deposition of materials onto a surface from gaseous precursors, enabling the growth of thin films or nanoparticles. Bottom-up techniques are particularly advantageous for fabricating highly uniform and functional nanomaterials, such as nanoparticles and nanorods, which can be used to deliver nutrients or bioactive agents to plants in agricultural applications [22].

### 2.2. Soft Lithography, Nanoimprinting, and Microfluidics

Soft lithography is widely used for transferring micro- and nanoscale patterns onto surfaces. It involves the formation of a mold using an elastomeric material, typically polydimethylsiloxane (PDMS), which is used to imprint patterns onto substrates, such as silicon, glass, and plastic [23]. Soft lithography offers several advantages, including low cost, flexibility of pattern design, and the applicability to various substrates [24]. This technique is particularly useful in agricultural research for fabricating microfluidic devices and nanopatterned surfaces that can influence microbial behavior [25]. Soft lithography can be used to develop interfaces that mimic natural root surfaces for promoting microbial colonization and modulating microbial communication, thereby enhancing plant growth and stress resistance [26].

Nanoimprinting, a recently developed technique, is cost-effective and provides high throughput for generating high-resolution nanoscale patterns [27], unlike traditional photolithography. This technique involves pressing a mold with nanoscale features onto a substrate to transfer a pattern [28]. Nanoimprinting can be applied to the production of nanostructured surfaces for seed coatings, microbial inoculants, and drug delivery systems, all of which have the potential to improve plant–microbe interactions and enhance agricultural productivity [29]. The precision afforded by nanoimprinting yields highly controlled features, essential for tailoring plant–microbe interactions at the molecular level [30].

Microfluidics is another critical nanofabrication technology, involving the manipulation of small volumes of fluids within micro-sized channels [31]. These devices allow researchers to generate controlled environments for studying microscale plant–microbe interactions, facilitating the observation of real-time microbial behavior [32]. Microfluidic systems are particularly useful for simulating the rhizosphere environment, in which plants and microbes interact under various conditions. In agricultural applications, microfluidics can be used to design lab-on-a-chip devices for the high-throughput screening of microbial interactions with plant roots or to monitor the release of exudates from plants in response to microbial colonization [32]. Table 1 compares these technologies in terms of their applications, advantages, and limitations regarding plant–microbe interactions.

### 2.3. Nanoparticle Synthesis: Green vs. Chemical Methods

**Figure 2:**
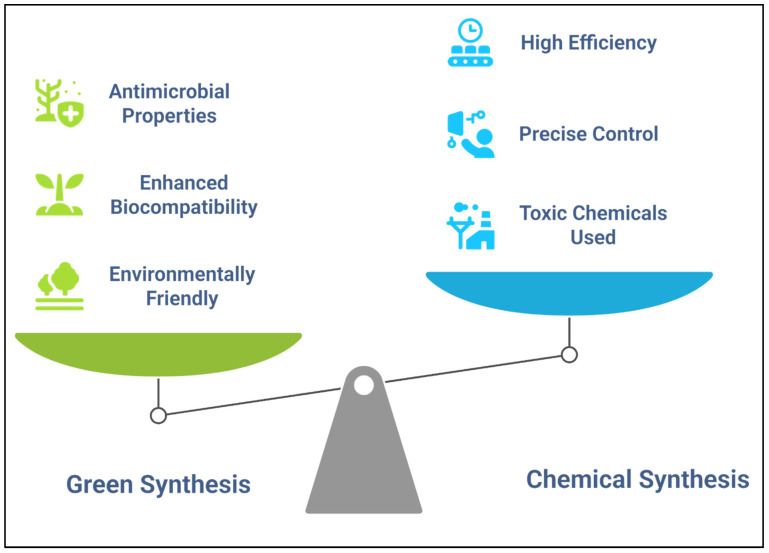
Comparison between green and chemical synthesis methods for synthesizing nanoparticles.

Nanoparticle synthesis can be used to develop delivery systems for bioactive agents in agriculture. Two primary methods of nanoparticle synthesis are green and chemical syntheses [39] (Figure 2). Green synthesis uses biological agents, such as plant extracts, microorganisms, or enzymes, to reduce metal salts into nanoparticles and avoids the use of toxic chemicals, thereby reducing environmental impact [40]. Green-synthesized nanoparticles are often highly biocompatible, which is essential for agricultural applications in which the impact on soil microbiota and plant health must be minimized [41]. For example, silver and gold nanoparticles synthesized using plant extracts have been shown to possess antimicrobial properties that can be applied to enhance plant protection against pathogens [42].

Chemical synthesis, in contrast, involves the use of chemical precursors to synthesize nanoparticles, often via processes such as chemical reduction, sol–gel synthesis, and hydrothermal methods [43]. Although chemical methods are highly efficient and allow for the precise control over particle size and shape, toxic chemicals that can negatively impact the environment are often used [40]. Despite these concerns, chemical synthesis remains a widely used technique for synthesizing nanoparticles with specific properties, such as high surface area or controlled release capabilities, useful in agricultural settings [44]. Nanoparticles synthesized via chemical methods are commonly used to develop controlled-release fertilizers, pesticides, and other agrochemicals, improving efficiency and reducing environmental contamination [45].

The choice between green and chemical syntheses depends on the application, the desired properties of the nanoparticles, and environmental considerations [46]. Both methods offer unique advantages and can be tailored to satisfy the requirements of agricultural nanotechnology, in which nanoparticles are used to deliver nutrients, protect plants from pests, and improve microbial inoculation efficiency [47].

### 2.4. Electrospinning and Nanocoating

Electrospinning is widely used for producing nanofibers from a polymer solution under a high applied voltage [48]. When an electric field is applied, the polymer solution forms a charged jet that solidifies into fine nanofibers when ejected from a nozzle [49]. The diameters of the resulting fibers are typically in the range of tens of nanometers to micrometers, rendering these fibers ideal for applications that require high surface areas and small scales. Additionally, these fibers are highly porous and biodegradable, which renders them suitable for a variety of agricultural applications (Table 2).

In agriculture, electrospun nanofibers are primarily used in controlled release systems, biosensors, and coatings [50]. The high surface areas and porosities of electrospun fibers allow for the encapsulation of substances such as fertilizers, pesticides, and microbial inoculants, which can be released slowly over time, improving efficiency and reducing environmental impact [51]. Controlled release systems prepared from electrospun fibers ensure that biofertilizers or biopesticides are gradually released to interact with plant roots or soil microbes [51]. This enhances nutrient availability while reducing the leaching or volatilization of these substances, which can otherwise cause environmental pollution.

Additionally, electrospun nanofibers are used to fabricate biosensors for the real-time monitoring of environmental factors such as soil moisture, temperature, and pH levels [52]. These biosensors, which benefit from the high surface area and sensitivity of electrospun materials, can monitor plant–microbe interactions, clarifying the status of the plant and the microbial communities around the plant. This can enable farmers to optimize inputs and monitor crop health in real time, allowing for precision farming [53].

By contrast, nanocoatings involve applying thin layers of nanomaterials to surfaces to modify surface properties, such as to enhance microbial adhesion and protect seeds from pathogens [54]. These coatings are applied to various surfaces, including plant seeds, agricultural tools, and packaging materials, to improve interactions between plants and microbes. Nanocoatings on seeds, for example, can substantially enhance microbial adhesion, promoting the colonization of beneficial microbes such as rhizobia, mycorrhizae, and PGPR, which can aid in nutrient uptake, disease resistance, and overall plant growth. Moreover, these coatings protect against pathogens, reducing the requirement for chemical pesticides [55].

Furthermore, nanocoatings are useful for protecting seeds from environmental stress and preventing pathogen invasion. Certain nanomaterials have intrinsic antimicrobial properties that act as barriers against seedborne diseases, improving seedling survival and promoting early plant growth [56]. Nanocoatings on fertilizers allow for the controlled release of nutrients, reducing excess runoff and improving nutrient efficiency [57]. This approach can minimize the environmental impact of fertilizers by ensuring that they are available to plants when required, preventing waste and reducing the risk of eutrophication.

**Table 2:** Applications of electrospinning and nanocoatings in agriculture.

Category	Application	Description	References
Seed Protection	Electrospun seed coatings	Electrospun nanofibers are used as seed coatings to protect seeds and enable the controlled release of agrichemicals, improving seedling development and crop protection	[58]
	Nanocoatings for seed protection	Nanocoatings applied to seeds enhance microbial adhesion, promote plant growth, and protect against pathogens	[59]
	Biodegradable nanofiber coatings for seeds	Coatings prepared using biodegradable nanofibers control the release of agrochemicals while enhancing seedling growth and minimizing environmental harm	[50]
Agrochemical Delivery	Agrochemical carriers	Nanofibers fabricated via electrospinning are used to encapsulate agrochemicals, improving controlled release and reducing environmental impact	[51]
Other Agricultural Uses	Biocompatible nanofiber membranes	Nanofibers provide a biocompatible porous membrane for storing seeds for protection while gradually releasing substances	[60]
	Antimicrobial nanocoatings for agricultural tools	Nanocoatings with antimicrobial properties are applied to agricultural tools and equipment to reduce contamination and improve tool longevity	[61]
	Microbial complex nanocoatings on seeds	Nanocoatings formed using electrospinning incorporate microbial complexes to enhance the interactions between plants and beneficial microbes	[62]
	Multilayer nanocoatings for crop protection	Multilayer nanocoatings, such as chitosan/lignin with silver nanoparticles, protect seeds and plants from pathogens	[63]
	Edible nanoencapsulation for food applications	Nanoencapsulation technology for food coatings and preservation, improving shelf life and food safety	[64]

## 3. Nanoengineered Delivery Systems

Nanoengineered delivery systems have emerged as transformative tools in agriculture, especially in terms of enhancing the efficacy and efficiency of microbial inoculants, biofertilizers, biopesticides, and plant growth regulators [13]. These systems use nanotechnology to improve the delivery, stability, and controlled release of bioactive compounds, ultimately optimizing plant–microbe interactions. The use of nanotechnology offers several advantages over traditional delivery methods, including enhanced bioavailability, targeted delivery to plant roots, and the ability to regulate the release of nutrients or active substances over time [65]. This section explores the revolutionization of agricultural practices owing to the use of nanoformulations in terms of improving microbial inoculation, biofertilizer performance, and biopesticide applications.

### 3.1. Nanoformulations for Microbial Inoculants

Microbial inoculants, including beneficial bacteria, fungi, and other microorganisms, are commonly used to enhance plant growth, improve soil health, and protect plants from pathogens [66]. However, the effectiveness of these inoculants is often limited by their stability, viability, and the ability to survive under harsh soil conditions. The use of nanoformulations encapsulating microbial inoculants in nanoparticles is a solution to these challenges, protecting the microorganisms from environmental stresses such as temperature fluctuations, desiccation, and ultraviolet (UV) radiation [67]. Moreover, these nanoformulations enhance the shelf lives of microbial inoculants, increasing the viability of long-term storage and application.

By encapsulating microbes in nanoparticles, the rate of microbial release can be controlled, ensuring that the inoculants are delivered directly to the plant roots at optimal concentrations over a sustained period [68]. Additionally, nanoparticles can be engineered to target specific areas in the soil or on plant roots, improving the efficiency of microbial colonization and promoting plant growth. For instance, nanoparticles made of biocompatible materials such as chitosan or alginate can be used to coat beneficial bacteria such as *Azospirillum* or *Rhizobium*, ensuring their effective colonization in the rhizosphere [69]. The application of nanoformulations is particularly advantageous in agricultural systems, such as in high-value crops or in soil environments, in which precision and efficiency are critical and nutrient availability is limited [70].

For instance, chitosan nanoparticles encapsulating *Rhizobium leguminosarum* have significantly improved nodulation and nitrogen fixation in *Phaseolus vulgaris*, enhancing plant nitrogen content and biomass [71,72]. Similarly, alginate-based nanoformulations of *Azospirillum brasilense* have shown improved colonization and root development in wheat under saline stress [13,73]. In another example, silica nanoparticles used for the delivery of *Bacillus megaterium* facilitated controlled microbial release over a 15-day period, enhancing phosphorus uptake and yield in rice [74,75]. This mechanism of controlled delivery via nanomaterials is highlighted in recent comprehensive reviews that emphasize the advantages of nanoencapsulation for extended microbial activity in soil ecosystems [76]. Furthermore, nanogels encapsulating mycorrhizal fungi have enhanced colonization in tomato roots, improving fruit quality and drought resistance [77,77]. These examples highlight the practical application of nanoformulations in real-world agricultural systems, especially under stress conditions where traditional inoculants are less effective.

### 3.2. Controlled Release of Biofertilizers and Biopesticides

A significant challenge in agricultural practices is the inefficiency and environmental impact associated with the application of chemical fertilizers and pesticides. Traditional fertilizers and pesticides often degenerate rapidly, leach, or evaporate, reducing their effectiveness and harming the environment via water contamination and soil degradation [78]. Controlled-release systems using nanotechnology can effectively address these issues by ensuring the slow, sustained release of biofertilizers, biopesticides, and other agrochemicals. This approach improves nutrient uptake by plants and minimizes the loss of active ingredients to the environment [79].

**Figure 3:**
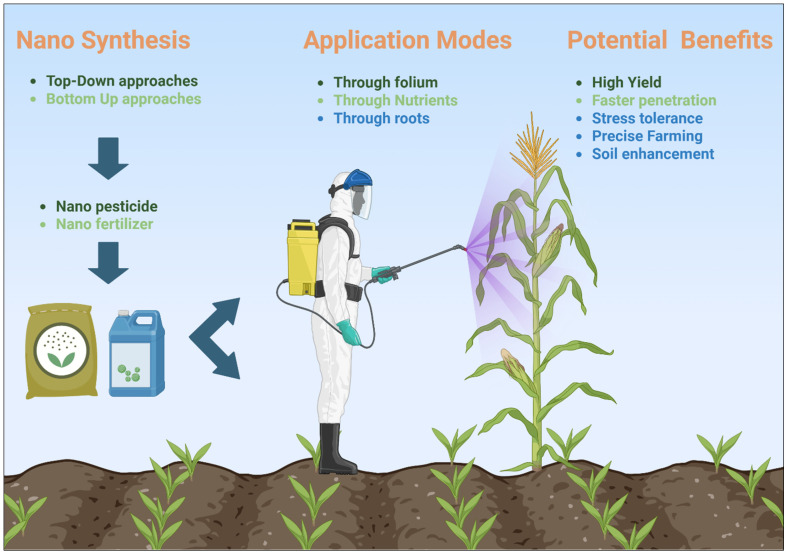
Synthesis application modes and benefits of nanofertilizers and nanopesticides.

Controlled-release systems can be designed using various nanomaterials, such as polymer nanoparticles, lipid-based carriers, and mesoporous silica nanoparticles [80]. These nanocarriers encapsulate active ingredients and protect them from premature degradation, gradually releasing these ingredients over time. For example, nanofertilizers, which release nutrients in response to specific environmental triggers (such as pH or temperature), ensure that plants receive a steady supply of essential nutrients throughout their growth cycle [81] (Figure 3). Similarly, the controlled release of nanoencapsulated biopesticides continuously protects against pests while reducing the frequency and amount of pesticide applications. The controlled release of biofertilizers and biopesticides reduces the environmental impact of agrochemicals by minimizing runoff and enhances the effectiveness of these products [82].

### 3.3. Nanocarriers for Rhizobia, Mycorrhizae, and PGPR

Nanocarriers have shown great potential in improving the delivery of beneficial microbes such as rhizobia, mycorrhizae, and PGPR to plant roots. These microbes enhance nutrient uptake, improve plant growth, and increase resistance to environmental stresses [83]. However, the survival and colonization of these beneficial microbes in the soil can be challenging owing to factors such as competition with indigenous microbial populations, environmental stressors, and poor soil conditions [84]. Nanocarriers, such as nanoparticles, nanogels, and nanofibers, can be used as protective delivery systems that improve the stability and viability of these beneficial microbes and enhance the ability of these microbes to effectively colonize plant roots (Table 3).

Nanocarriers can be engineered to enable the controlled release of microbes, ensuring that the microbes reach the roots in optimal concentrations at the optimal time [85]. For example, rhizobia, responsible for nitrogen fixation in legumes, can be encapsulated in biodegradable nanocarriers, enabling the sustained release of these microbes into the root zone. This method enhances the symbiotic relationship between the plant and the rhizobia, improving nitrogen uptake and reducing the requirement for synthetic fertilizers [86]. Similarly, mycorrhizal fungi, which form symbiotic relationships with plant roots to enhance phosphorus uptake, can be delivered using nanocarriers that protect the fungi from environmental stress and promote the successful colonization of the root system [87]. PGPR, which promote plant growth by producing plant hormones or suppressing plant pathogens, can be effectively delivered using nanocarriers, ensuring that these PGPR reach the plant roots and impart beneficial effects [83].

Several recent studies have provided specific examples demonstrating the practical effectiveness of nanoparticle-based treatments in agricultural contexts. For instance, silica nanoparticles have been utilized to encapsulate and deliver *Rhizobium leguminosarum* to pea plants (*Pisum sativum*), significantly enhancing microbial colonization, nodule formation, and nitrogen fixation efficiency [88,89,90]. In another example, chitosan-based nanogels have been successfully used to deliver arbuscular mycorrhizal fungi such as *Glomus intraradices* to maize plants (*Zea mays*), improving phosphorus uptake, plant biomass, and tolerance to drought stress [91,92]. Furthermore, carbon nanotube-mediated delivery of rhizobia and mycorrhizal fungi has been reported in soybean (*Glycine max*), *Zea Mays*, and barley (*Hordeum vulgare*), markedly increasing nutrient uptake and overall plant growth [88]. Lipid-based nanocarriers containing plant growth-promoting rhizobacteria (PGPR) such as *Pseudomonas fluorescens* have also been effectively applied to tomato plants (*Solanum lycopersicum*), enhancing root colonization, nutrient absorption, and resistance to soil pathogens [93,94,95]. These detailed cases underscore the substantial potential of nanocarriers to improve the effectiveness of beneficial microbial inoculants, plant health, and crop productivity under field conditions.

**Table 3:** Summary of the nanocarriers used for delivering rhizobia, mycorrhizae, and PGPR.

Nanocarrier Type	Microbe Type	Application	Functionality	References
Nanoparticles	Rhizobia	Encapsulation of rhizobia for sustained release to improve nitrogen fixation in legumes and reduce synthetic fertilizer use	Enhancing nitrogen fixation by maintaining a steady delivery of rhizobia to plant roots, thereby improving the symbiotic relationship between the plant and the microbe	[96]
Nanoemulsions	PGPR	Delivery system for PGPR that promotes plant growth by enabling the controlled release of beneficial microbes in the root zone	PGPR enhance plant growth via hormone production and pathogen suppression; nanoemulsions enable sustained release for consistent microbial activity	[83]
Bio-nanofertilizers	PGPR, microalgae	Functionalized nanoparticles with PGPR and microalgae for enhancing nutrient uptake and promoting plant growth	Improves nutrient availability and plant growth via synergistic effects of PGPR and microalgae; nanoparticles protect microbes from environmental stress	[93]
Nanofibers	Mycorrhizae	Delivery system for mycorrhizal fungi to promote phosphorus uptake and successfully colonize plant roots	Mycorrhizal fungi enhance nutrient uptake, particularly phosphorus; nanofibers provide physical protection and the controlled release of fungi to the root system	[97]
Polymeric nanoparticles	PGPR	Use of nanoparticles for the controlled delivery of PGPR, enhancing plant growth and resilience to drought conditions	Nanoparticles provide a stable environment for PGPR, promoting plant health under adverse conditions	[98]
Nanoparticles (zinc and iron)	PGPR, mycorrhizae	Zinc and iron nanoparticles functionalized with PGPR for enhancing plant growth and mycorrhizal colonization	These nanoparticles supply essential micronutrients to plants, whereas PGPR and mycorrhizae improve nutrient uptake and plant health	[99]
Silica nanoparticles	Rhizobia, PGPR	Delivery of rhizobia and PGPR using silica nanoparticles to improve microbial colonization and stress resistance in plants	Silica nanoparticles improve microbial survival under stressful environmental conditions by providing stability and protection	[100]
Chitosan-based nanogels	Mycorrhizae, PGPR	Chitosan nanogels for delivering mycorrhizal fungi and PGPR to increase soil fertility and promote plant growth	Chitosan-based nanogels are biodegradable and provide a slow-release mechanism for both mycorrhizal fungi and PGPR, ensuring sustained microbial activity in the root zone	[13]
Carbon nanotubes	Rhizobia, mycorrhizae	Delivery of rhizobia and mycorrhizae using carbon nanotubes to increase microbial efficacy and plant growth	Carbon nanotubes increase the efficiency of microbial delivery to plant roots, enhancing nutrient uptake and plant growth	[101]
Lipid-based nanocarriers	PGPR	Lipid-based nanoparticles for the controlled delivery of PGPR in the rhizosphere to enhance plant growth and resilience	Lipid carriers offer targeted delivery and increase microbial colonization by forming a protective barrier that prevents degradation	[102]

## 4. Nanostructured Surfaces for Root–Microbe Interactions

Nanostructured surfaces can potentially enhance plant–microbe interactions by mimicking natural plant root surfaces, which are key sites for microbial attachment and colonization. The ability to engineer materials at the nanoscale can generate surfaces with precise topographies, charges, and surface energies that can influence microbial behavior [10]. These nanostructured materials enhance the attachment and promote the growth and activity of beneficial microbes, such as rhizobia and mycorrhizae. The integration of nanotechnology into the design of surfaces that facilitate plant–microbe interactions is essential for improving plant health, growth, and resilience and for advancing sustainable agricultural practices [103]. Nanostructured surfaces can be designed to improve the colonization of plant roots by beneficial microbes, thereby enhancing nutrient uptake, disease resistance, and stress tolerance.

### 4.1. Engineering Root-Mimetic Interfaces

The plant root system constitutes a complex and dynamic environment for microbial communities. This system features unique structural and chemical properties that enable the adhesion of microorganisms, forming a crucial interface for nutrient exchange, growth, and symbiotic relationships [104]. Engineering root-mimetic interfaces using nanostructured materials replicates these natural root properties, enhancing microbial attachment and promoting beneficial interactions [105]. These nanostructured interfaces can be designed to mimic physical and chemical cues, such as surface roughness, charge, and hydrophobicity, found on root surfaces, influencing microbial adhesion and biofilm formation [106]. By incorporating specific nanomaterials, the surface properties of these interfaces can be tuned to attract desired microorganisms or inhibit the growth of harmful pathogens [107].

For instance, nanomaterials such as carbon nanotubes, silica nanoparticles, and nanofibers can be used to synthesize surfaces with controlled roughness at the nanoscale [108]. These surfaces enhance microbial attachment by increasing the surface area, establishing beneficial microbial communities. Additionally, the surface charge and chemical composition can be tailored to promote the growth of specific microbes, such as nitrogen-fixing rhizobia or PGPR, while discouraging the colonization of pathogenic microbes [109]. Furthermore, root-mimetic interfaces modulate the physical environment, including water retention and nutrient availability, of the plant–microbe interface, further enhancing the efficiency of microbial colonization [110]. The application of these engineered interfaces in agriculture can be used to develop sustainable, eco-friendly farming practices, in which microbial inoculants can better establish themselves in soil, improving crop yield and soil health [111].

### 4.2. Nanostructured Seed Coatings for Enhanced Microbe Colonization

**Figure 4:**
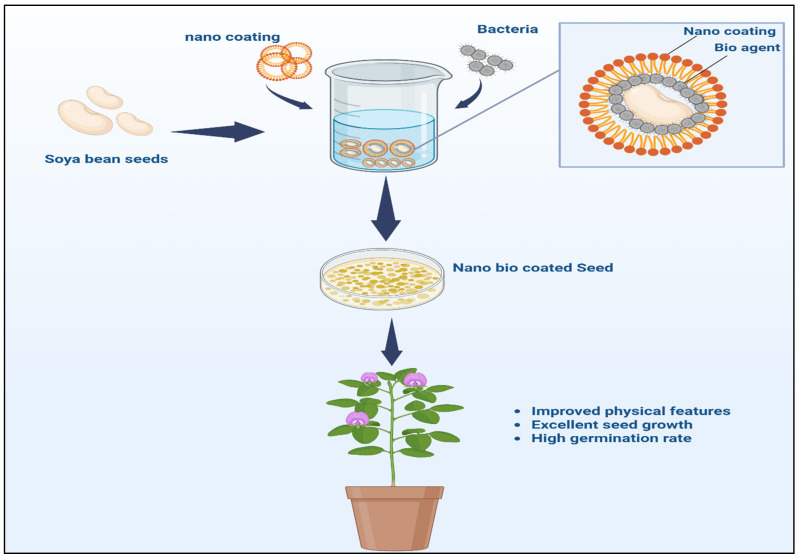
Illustration of the nanostructured seed coatings and their interactions with microorganisms to facilitate microbial adhesion and promote seedling growth.

The use of seed coatings is an effective strategy for improving microbial colonization and enhancing plant growth. Traditional seed treatments often rely on chemical fertilizers and pesticides, which can adversely impact the environment [112]. The use of nanostructured coatings, however, is a sustainable approach that yields a controlled environment that promotes microbial adhesion and protects seeds from pathogens [113]. These coatings can be engineered to deliver beneficial microbes directly to the seed surface, ensuring that the microbes are near the emerging plant roots. The nanoscale features of these coatings improve the adhesion of microbial inoculants, ensuring that beneficial microorganisms are well-established in the early stages of plant growth [113] (Figure 4).

Nanostructured seed coatings can be prepared using a variety of materials, including biopolymers, nanoclays, and biodegradable polymers, for encapsulating microorganisms such as mycorrhizae, rhizobia, and PGPR [114]. These coatings protect microbes from desiccation, UV radiation, and other environmental stressors, thereby improving the survival rates of these microbes. As the seed germinates and grows, the nanostructured coating gradually releases the microorganisms, enabling sustained microbial colonization as the plant develops [112]. This controlled release of microbes can improve the efficiency of nutrient uptake, disease resistance, and plant health. Furthermore, these coatings can physically protect against pathogens, reducing the need for chemical treatments and minimizing the environmental footprint of conventional seed treatments [115].

Specific examples illustrate the effectiveness of nanostructured seed coatings in agricultural applications. For instance, chitosan-based nanocoatings incorporating beneficial bacteria such as *Azospirillum brasilense* and *Rhizobium leguminosarum* have demonstrated significant improvements in wheat (*Triticum aestivum*) and bean (*Phaseolus vulgaris*) seedling growth, nutrient uptake, and tolerance to environmental stress [112,116,117,118]. Multilayer nanocoatings composed of chitosan and lignin, incorporated with silver nanoparticles, have been effectively applied to maize (*Zea mays*) and soybean (*Glycine max*) seeds, offering robust protection against fungal pathogens, such as *Fusarium* and *Rhizoctonia* species [119,120]. These examples clearly demonstrate the potential of nanostructured seed coatings to significantly enhance microbial colonization, promote plant health, and offer sustainable alternatives to conventional chemical treatments [121].

### 4.3. Influence of Surface Charge, Porosity, and Hydrophobicity

The physical and chemical properties of nanostructured surfaces, particularly surface charge, porosity, morphology and hydrophobicity, determine the adherence to and colonization of these surfaces by these microorganisms (Table 4). These properties strongly influence microbial interactions in agricultural systems, especially when nanomaterials are employed to promote plant–microbe symbiosis [122,123]. By understanding the effect of these properties on microbial behavior, the design of nanomaterials for specific agricultural applications can be optimized, enhancing microbial colonization and, in turn, plant growth and soil health.

Surface charge is one of the most influential factors affecting microbial adhesion. Surfaces can either be positively or negatively charged, influencing the electrostatic interactions between the surface and microorganisms, which are also typically charged [124]. Positive surface charges tend to attract negatively charged microbial cells owing to electrostatic forces, facilitating the adhesion and colonization of beneficial microbes such as rhizobia and mycorrhizae [125,126]. These microbes are crucial for nutrient cycling and the symbiotic relationships with plant roots. The adhesion of such microbes can be beneficial for enhancing plant growth and soil health. Conversely, a negatively charged surface may either repel or attract certain types of microorganisms, depending on their charge, forming a dynamic microbial environment [126,127]. In some applications, such as controlling pathogenic microorganisms, negatively charged surfaces may prevent the adhesion of harmful microbes, generating a natural defense mechanism.

Porosity—the presence of pores or voids within a material—considerably affects microbial colonization. Nanomaterials with high porosity possess large surface areas, which can increase microbial adhesion [128,129]. This is particularly advantageous when designing materials to promote the colonization of beneficial microbes, such as PGPR, which can improve nutrient uptake, disease resistance, and plant growth. Moreover, high porosity enhances the retention of water, nutrients, and other beneficial substances, forming an ideal microenvironment for microbial activity [130]. Several synthetic methods can be used to manipulate porosity in nanomaterials, such as sol–gel processing, electrospinning, and templating. For example, sol–gel processing is widely used to create silica-based nanoparticles with varying porosity, which are beneficial for microbial adhesion and controlled release of inoculants [131,132]. Similarly, electrospinning is used to produce nanofibers with controlled pore sizes, enhancing the encapsulation of microbes and their slow release into the soil [133,134]. Although high porosity generally promotes microbial colonization, porosity must be controlled to avoid excess microbial growth, which could generate unwanted effects, such as nutrient competition and overgrowth that hinders plant development. By contrast, materials with low porosity may not possess enough surface area for optimal microbial colonization but can be useful for controlled release applications in which minimal microbial interaction is required [135]. For example, low-porosity nanomaterials are often used in slow-release fertilizers or pesticide delivery systems, in which regulating the release of active substances without encouraging excessive microbial colonization is essential [136].

However, the morphology of nanomaterials is equally crucial in influencing microbial colonization. The shape, size, and surface roughness of nanomaterials can greatly affect how microorganisms adhere to these surfaces. Nanoparticles with higher surface roughness provide more physical sites for microbial attachment, facilitating better colonization. For instance, nanorod-shaped materials have been shown to increase the attachment of beneficial bacteria such as *Pseudomonas fluorescens* on plant roots [137]. Similarly, nanotube-shaped nanomaterials can provide increased surface area and enhanced microbial colonization efficiency, particularly for *Rhizobium* species in legumes [88,138]. Studies also show that nanomaterials with controlled surface roughness, achieved through methods like laser ablation or electrospinning, increase microbial adhesion by providing greater surface area for attachment. The rougher the surface, the greater the number of points at which microbial cells can attach, which improves colonization and functional activity, such as nutrient exchange and pathogen resistance. For example, laser-textured surfaces have been shown to reduce bacterial adhesion by increasing surface roughness, which in turn inhibits biofilm formation [139,140]. Additionally, nanoscale surface roughness in titanium and titanium alloy surfaces has been demonstrated to improve microbial rejection and bioactivity by promoting increased wettability and cell attachment while reducing bacterial adhesion [141,142].

Hydrophobicity, or the tendency of a surface to repel water, is another critical property that affects microbial attachment. Nanostructured materials that exhibit hydrophobic characteristics tend to repel water, which can inhibit the attachment of water-loving microbes and limit microbial colonization [143,144]. These materials can be particularly useful for reducing microbial interactions or when water retention is undesirable. For instance, hydrophobic coatings can be used on seeds or plant surfaces to protect these surfaces from excess moisture, which may otherwise encourage the growth of harmful pathogens [112]. Additionally, hydrophobic surfaces are useful in applications in which microbial contamination must be reduced or microbial biofilms that could otherwise interfere with plant growth must be controlled [145].

In contrast, hydrophilic surfaces, which attract and retain water, generate an ideal environment for the adhesion of water-loving microbes [143]. These surfaces facilitate the colonization of beneficial microorganisms, such as mycorrhizae and rhizobacteria, that thrive in moist environments. Moreover, hydrophilic surfaces can promote water and nutrient retention around plant roots, enhancing the availability of resources to the plants and microbes. In agricultural systems, hydrophilic nanomaterials are commonly used in seed coatings or plant root interfaces to establish microbial communities that support plant growth, nutrient uptake, and disease resistance [143].

The interplay between surface charge, porosity, morphology, and hydrophobicity determines the interactions between nanomaterials and microorganisms in agricultural settings. By understanding and controlling these factors, nanomaterials that either promote or limit microbial colonization in a controlled and purposeful manner can be designed. Tailoring nanomaterials to specific agricultural needs allows for the optimization of plant–microbe interactions, enhancing crop productivity, soil health, and sustainable agricultural practices.

**Table 4:** Comparison of surface properties and their impacts on microbial colonization.

Surface Property	High Value (Effect)	Low Value (Effect)	References
Surface charge	Positive charge attracts negatively charged microbes, enhancing microbial adhesion (e.g., PGPR and mycorrhizae), facilitating nutrient cycling and plant growth	Negative charge repels some microbes, useful for preventing pathogen adhesion and can generate dynamic microbial environments for controlling harmful microbes	[126]
Porosity	High porosity increases surface area, promoting microbial colonization, enhances microbial activity, supports the retention of nutrients and moisture, and improves soil health and plant growth	Low porosity reduces surface area, limiting microbial colonization, but is useful for controlled release applications such as slow-release fertilizers and pesticide delivery systems	[129,146]
Hydrophobicity	Hydrophobic surfaces inhibit water-loving microbes, limiting microbial colonization. Useful for dry conditions or controlling microbial biofilms and pathogen growth on plant surfaces	Hydrophilic surfaces enhance microbial adhesion and water retention, promoting colonization of beneficial microbes, especially in moist environments such as plant roots	[144]
Surface roughness	High surface roughness increases available surface area for microbial attachment, supporting increased microbial colonization, especially for symbiotic microbes	Low surface roughness limits the available area for microbial attachment and may reduce microbial colonization potential	[106]
Surface functionalization	Functionalized surfaces can enhance or inhibit microbial attachment. For example, adding hydrophilic or hydrophobic functional groups allows for targeted microbial interactions (e.g., promoting beneficial microbe colonization or controlling pathogen biofilm formation)	Lack of surface functionalization can cause passive microbial attachment, causing inefficient or unintended microbial colonization	[147]
Environmental factors (pH and ionic strength)	Environmental conditions such as pH and ionic strength can influence the interactions between surface charge and microbial adhesion, promoting or reducing microbial attachment depending on the conditions	Inconsistent environmental factors can alter the effectiveness of surface charge and other properties, potentially reducing microbial colonization and unpredictable results	[148]

## 5. Biosensors and Microfluidics for Interaction Monitoring

The dynamic and complex nature of plant–microbe interactions requires real-time monitoring to clarify the physiological responses of plants and the behaviors of microbes in the rhizosphere. Biosensors and microfluidic devices are invaluable tools in this regard, enabling the acquisition of high-resolution data on microbial activity, root exudate profiles, and other key factors influencing these interactions [32]. These technologies present unprecedented opportunities to study microbial colonization, monitor the release of bioactive compounds from plant roots, and observe microbial responses to environmental cues. By integrating biosensors and microfluidic systems into agricultural research, scientists can study the mechanisms that underpin beneficial plant–microbe symbioses in depth, increasing the efficiency and improving the targeted approaches for enhancing crop health and productivity [149,150].

### 5.1. Real-Time Monitoring of Microbial Colonization

**Figure 5:**
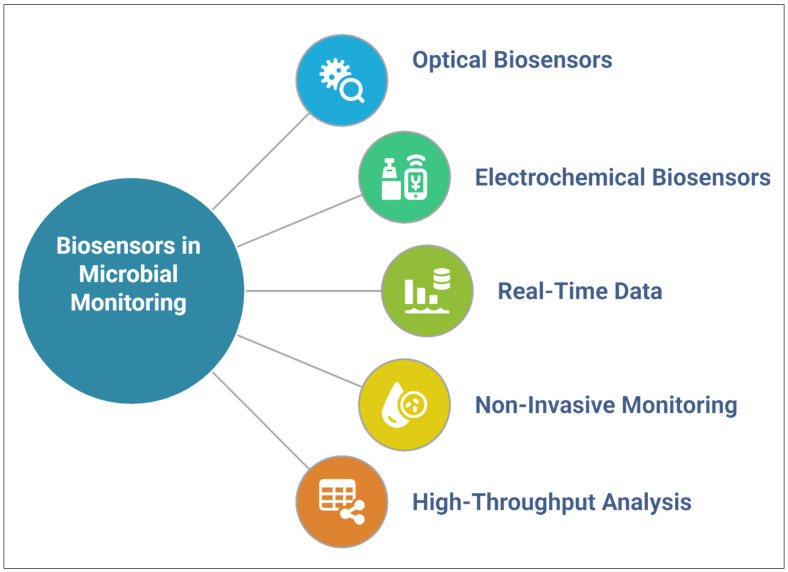
Applications of biosensors for monitoring microbial colonization.

Biosensors provide real-time data on microbial colonization, allowing researchers to monitor the dynamics of microbial communities in response to various environmental factors, such as nutrient availability, temperature, and soil moisture [151] (Figure 5). Traditional methods for monitoring microbial colonization, such as plating techniques and microscopy, are time-consuming and often lack the resolution required to study microbial interactions in dynamic environments [151,152]. However, biosensors offer a non-invasive and high-throughput alternative that can provide continuous, real-time data on microbial populations. These sensors can detect changes in microbial abundance, activity, and health, providing valuable insights into the interaction of microbes with plant roots and the surrounding soil environment [151].

Biosensors used in these applications can be based on various principles, such as optics, electrochemistry, and fluorescence. For example, optical biosensors can detect changes in microbial activity by measuring shifts in light absorption or fluorescence emissions, indicative of metabolic processes occurring within the microbial community [153]. By contrast, electrochemical biosensors can measure changes in the electrical properties of microbial cells, such as impedance and conductivity, as the cells grow and interact with plant roots [154]. These biosensors are particularly useful for monitoring the establishment of beneficial microorganisms, such as nitrogen-fixing rhizobia or PGPR, in the rhizosphere [153]. By tracking microbial colonization in real time, researchers can further their understanding of the contribution made by these microbes to plant health and the influence of environmental factors on the establishment and activity of these microbes.

### 5.2. Lab-On-A-Chip for Profiling Root Exudates

**Figure 6:**
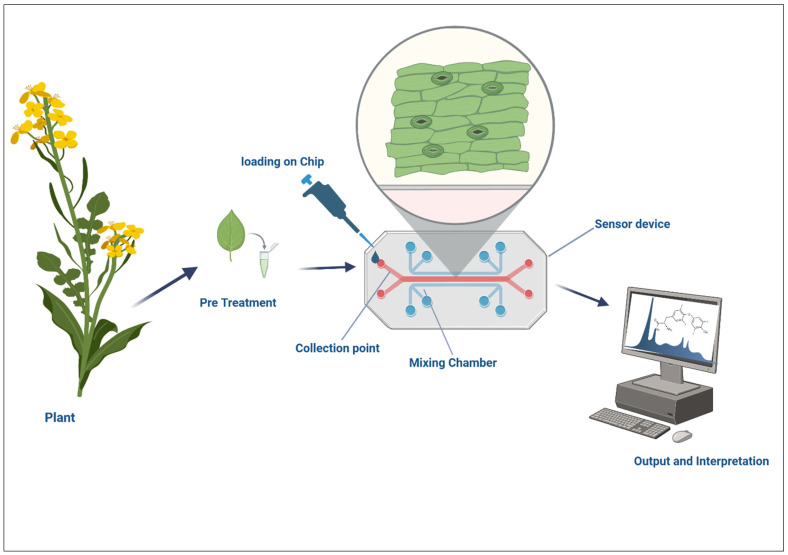
Schematic of an LOC device for profiling root exudates and detecting secondary metabolites.

Lab-on-a-chip (LOC) devices are another powerful tool for monitoring plant–microbe interactions, particularly for profiling root exudates [155]. Root exudates are a diverse group of compounds released by plant roots into the surrounding soil, influencing plant–microbe signaling, nutrient uptake, and pathogen defense [156] (Figure 6). These exudates include sugars, amino acids, organic acids, and various secondary metabolites, all of which can influence microbial behavior and community composition in the rhizosphere [157]. Profiling root exudates in real time and with high precision clarifies the biochemical signals exchanged between plants and microbes, uncovering the mechanisms underlying microbial recruitment and the establishment of beneficial symbioses [158].

LOC devices enable high-throughput, miniaturized analysis of root exudates, enabling the simultaneous detection of multiple metabolites at low concentrations. These devices consist of micro-sized channels and chambers that can simulate the rhizosphere environment, enabling the collection and analysis of root exudates under controlled conditions [159]. LOC systems can incorporate various detection methods, such as mass spectrometry, gas chromatography, and fluorescence sensing, to identify and quantify exudates released by plant roots. The small sizes and portability of LOC devices render them particularly useful for on-site, field-based analysis, in which researchers can monitor root exudate profiles under different environmental conditions and treatment regimens [160,161]. Additionally, the integration of LOC systems with biosensors enables continuous, real-time monitoring of root exudate dynamics, comprehensively determining the influence of plants on their surrounding microbial communities [162].

### 5.3. Nanosensors for pH, Reactive Oxygen Species, and Metabolite Detection

Nanosensors have emerged as a valuable tool for monitoring various environmental parameters during plant–microbe interactions, clarifying the physiological status of both plants and microorganisms in real time. These sensors enable the detection of crucial factors such as pH, reactive oxygen species (ROS), and metabolites, vital for understanding the dynamic processes that occur in the rhizosphere [162,163].

Notably, pH influences nutrient availability and microbial activity around plant roots. Plant roots release organic acids that alter soil pH, affecting microbial communities and nutrient solubility [164]. Nanosensors sensitive to pH changes often contain materials such as quantum dots, carbon-based nanomaterials, or metal oxide nanostructures [165]. These materials shift their optical or electrochemical properties based on pH fluctuations, allowing pH to be monitored precisely and continuously in real time. This capability is crucial for understanding the response of microbial populations to pH shifts, which can impact nutrient uptake and overall plant health [166].

ROS detection is equally important, as ROS are involved in plant defense mechanisms and microbial pathogenesis. Both plants and microbes produce ROS in response to stress, including pathogen attack and oxidative damage [167]. Nanosensors designed for ROS detection are made of materials such as graphene oxide or quantum dots that react with ROS, generating a detectable signal. By monitoring ROS levels, these sensors provide real-time data on oxidative stress, elucidating the balance between plant immune responses and microbial activity, particularly during pathogen infection and environmental stress conditions [168].

Metabolite detection allows the exchange of metabolites between plants and microbes, a process that influences nutrient cycling, signaling, and symbiotic relationships, to be monitored [169]. Nanosensors that can detect metabolites typically rely on molecularly imprinted polymers or enzymatic reactions that specifically bind to target metabolites, such as phytohormones, organic acids, and secondary metabolites produced by microbes. By detecting metabolites such as auxins and cytokinins, nanosensors provide valuable information regarding the influence of microbial communities on plant growth and the modulation of the metabolic responses of plants to microbes [170].

## 6. Environmental and Safety Considerations

As the use of nanotechnology in agricultural applications continues to advance, the environmental and safety implications associated with the use of nanomaterials in agricultural systems must be considered [171]. Although nanomaterials offer several advantages, such as improving the delivery of nutrients and enhancing plant–microbe interactions, the potential impact of nanomaterials on the environment and non-target organisms must be thoroughly determined [171]. The use of nanomaterials in agriculture introduces new challenges in terms of their persistence in the environment, interactions with soil ecosystems, and effects on plant and microbial health [172]. Consequently, research into the biodegradability and toxicity of these materials is critical to ensuring their safe and sustainable use in agricultural practices.

### 6.1. Biodegradability of Nanomaterials in Agricultural Systems

The biodegradability of nanomaterials is a key factor for determining their long-term environmental impact and sustainability in agricultural systems. Nanomaterials are considered to be safe for agricultural use if they break down into non-toxic, environmentally benign components over time, preventing the accumulation of harmful substances in soil and water systems [173]. Biodegradable nanomaterials can be engineered to degrade via natural processes, such as microbial activity, photodegradation, or hydrolysis, unlike conventional chemical fertilizers and pesticides, which may persist in the environment for extended periods. The rates and pathways of degradation depend on the chemical composition, size, surface properties, and shapes of the nanomaterials [174].

Biodegradable nanomaterials, such as biopolymers and nanoparticles synthesized using naturally occurring materials (e.g., starch, chitosan, or polylactic acid) [175] can be designed to degrade gradually over time, ensuring the controlled release of bioactive compounds and nutrients, thereby minimizing environmental harm [176]. For instance, nanoparticles synthesized using biodegradable polymers can deliver microbial inoculants or biofertilizers to plants, where these nanoparticles break down upon exposure to environmental conditions, reducing the requirement for frequent reapplications and minimizing environmental footprint [177]. The study of the biodegradation rates of nanomaterials is essential for assessing environmental persistence and determining safe application practices in agriculture. Researchers are designing nanomaterials that maintain functionality during intended use but degrade safely once the purpose of these nanomaterials has been served, ensuring minimal residual impact on soil health, water quality, and surrounding ecosystems [173,178].

### 6.2. Nanotoxicity to Soil Microbiota and Plants

**Figure 7:**
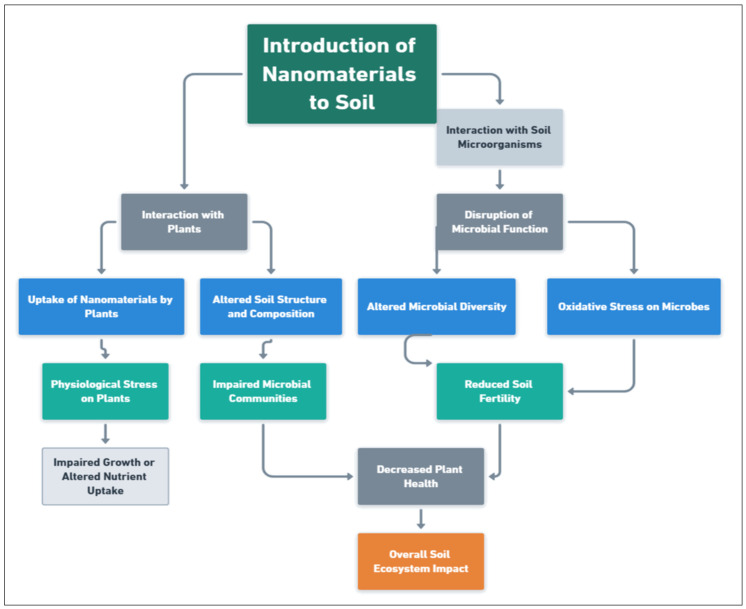
Possible nanotoxicity in the soil ecosystem.

Nanotoxicity is a growing concern as the widespread use of nanomaterials in agriculture may affect plants and soil microbiota, influencing soil health and fertility [179]. The potential risks posed by nanomaterials to soil organisms, including microorganisms, invertebrates, and plants, have still not been fully understood, necessitating further research to determine the mechanisms and extent of the toxicity of these materials [180] (Figure 7). Soil microorganisms, such as bacteria and fungi, are integral to nutrient cycling, organic matter decomposition, and the establishment of beneficial plant–microbe symbioses [181,182]. Introducing nanomaterials into soil systems may disrupt these vital processes by affecting microbial communities, either directly owing to toxicity or indirectly owing to alterations in soil structure and composition [183].

Nanotoxicity can manifest in various forms, depending on the type, size, shape, surface charge, and concentration of the nanomaterial. For example, nanoparticles with a high surface area or surface reactivity may interact with microbial cell membranes, disrupting cell function and causing oxidative stress. This can reduce microbial diversity and activity, potentially impairing soil fertility and plant health [184]. Similarly, nanomaterials may accumulate in plant tissues, causing physiological stress, impairing growth, and altering nutrient uptake. The toxicity of nanomaterials to plants is influenced by factors such as the types and exposure times of nanomaterials and environmental conditions, which can vary widely under agricultural settings [185].

To mitigate the potential risks of nanotoxicity, researchers are attempting to understand the interactions between nanomaterials and soil microorganisms and the corresponding uptake and transport within plants [186]. Such research is crucial for developing strategies to minimize toxicity, such as modifying the surface properties of nanoparticles to reduce reactivity or using less toxic materials for nanomaterial synthesis. Additionally, studies are exploring whether nanomaterials can positively affect soil microbiota by stimulating microbial growth or promoting beneficial microbial communities, offsetting any negative impacts [85]. As agricultural nanotechnology advances, establishing clear guidelines and regulatory frameworks will be essential to ensure the safe use of nanomaterials in farming practices to maintain soil health and safeguard plant and microbial ecosystems [173].

## 7. Challenges and Future Directions

Although nanotechnology has shown great promise in transforming agricultural practices, several challenges remain that must be addressed to achieve successful integration into field applications [187]. These challenges are associated with scalability, cost, and environmental safety, essential for ensuring that nanotechnology can be applied effectively and sustainably in real-world agricultural settings [188]. Moreover, combining nanotechnology with emerging technologies such as artificial intelligence (AI), synthetic biology, and the Internet of Things (IoT) offers exciting opportunities for enhancing precision agriculture and sustainable farming practices [189]. This section explores these challenges and the future directions for integrating nanotechnology into agriculture.

### 7.1. Integration of Nanotechnology into Field Applications

The integration of nanotechnology into field applications presents challenges, including the differences between laboratory-scale experiments and real-world agricultural conditions [190]. One of the primary challenges is related to the scalability of nanofabrication techniques. Although the laboratory-scale production of nanomaterials has been successful, scaling up these processes for large-scale agricultural applications remains complex. Nanomaterials must be produced in large quantities without compromising their performance, and this requires the manufacturing methods to be optimized to ensure uniformity, consistency, and cost-effectiveness. Additionally, the environmental impact of large-scale nanomaterial production must be carefully considered to avoid generating new environmental hazards [191,192].

Cost is another major barrier to the widespread adoption of nanotechnology in agriculture. Although nanomaterials offer numerous benefits, producing them is often expensive, especially when high-performance materials are required for specific applications, such as nanoencapsulated fertilizers or bioactive agents [193]. To overcome this challenge, efficient nanomanufacturing techniques that can reduce production costs are required. Researchers are working to identify cost-effective raw materials, develop low-cost synthesis methods, and improve the overall efficiency of production processes [194,195]. Furthermore, concerns exist regarding the potential environmental impact of nanomaterials introduced into agricultural ecosystems. Issues such as the persistence of these nanomaterials in soil, interactions with soil organisms, and potential toxicity to plants or microbes need to be thoroughly addressed to ensure that nanomaterials do not pose long-term risks to the environment [173].

### 7.2. Scalability and Cost-Effectiveness of Nanofabrication Techniques

Scalability and cost-effectiveness are crucial for the successful implementation of nanotechnology in agriculture. Current nanomanufacturing techniques, such as CVD and sol–gel synthesis, are often too expensive and inefficient for large-scale agricultural applications [196]. To overcome these challenges, manufacturing methods that are scalable and economically viable must be developed. A promising approach involves bottom-up nanofabrication techniques, which allow for the self-assembly of nanomaterials [18]. These techniques, such as self-assembly and nanoimprinting, can be more cost-effective and scalable than top-down techniques, which rely on expensive equipment and precisely controlled material removal.

Another area of focus is the development of sustainable materials that are both affordable and effective for agricultural use. For instance, using naturally abundant and biodegradable materials for nanoparticle synthesis can reduce the overall cost of production while ensuring environmental safety. Additionally, optimizing the processes involved in the synthesis and functionalization of nanomaterials can improve yield and reduce material costs. As nanotechnology continues to evolve, advancements in manufacturing technologies and materials will be critical for ensuring that nanomaterials can be produced at scale and cost-effectively, improving accessibility for widespread use in agriculture [173].

### 7.3. Synergies with AI, Synthetic Biology, and IoT in Agricultural Advancements

The future of agriculture involves the integration of nanotechnology with other advanced technologies, such as AI, synthetic biology, and IoT [197]. When combined with AI, nanotechnology can enable precision agriculture by providing real-time data on plant health, soil conditions, and microbial activity, allowing farmers to make data-driven decisions regarding efficient farming practices. AI algorithms can process large volumes of data collected from nanotechnology-based sensors, such as those used for monitoring soil moisture, nutrient levels, and microbial populations, to optimize farming practices and predict crop yields [198].

Synthetic biology, involving the design and engineering of biological systems, can further enhance the potential application of nanotechnology in agriculture [199]. By combining synthetic biology with nanotechnology, researchers can engineer microorganisms that can interact with nanomaterials to perform specific tasks, such as enhancing nutrient uptake, promoting plant growth, and providing pest resistance [10]. For example, genetically engineered bacteria or fungi may be used in conjunction with nanoparticle carriers to deliver nutrients or biocontrol agents directly to plant roots, improving the effectiveness of fertilizers and pesticides while reducing environmental impact [200].

The IoT plays a critical role in the future of smart farming by providing interconnected systems for monitoring and controlling agricultural processes [201]. Nanotechnology-based sensors can be integrated into IoT systems to enable the real-time monitoring of plant and soil conditions, enabling farmers to optimize resource usage and reduce waste [202]. For instance, IoT-enabled smart irrigation systems can use data from nanotechnology sensors to deliver optimal amounts of water and nutrients to crops, reducing water and fertilizer consumption [203]. The integration of these technologies will improve the sustainability and precision of farming practices, improving crop productivity while minimizing the environmental footprint of agriculture.

## 8. Conclusions

The use of nanotechnology is highly promising for transforming plant–microbe interactions, offering innovative solutions for enhancing agricultural productivity and sustainability. This review contributes to the field by providing a comprehensive overview of how nanomaterials can be integrated into agricultural systems to improve microbial inoculant stability, nutrient uptake, and the controlled release of biofertilizers and biopesticides. Additionally, the review highlights the role of nanostructured surfaces such as root-mimetic interfaces and nanocoated seeds in promoting microbial colonization, enhancing plant growth, and protecting crops from pathogens. The integration of biosensors and microfluidics for real-time monitoring of plant–microbe dynamics also provides significant advancements in understanding microbial behavior and plant–microbe signaling.

Despite these promising advancements, the widespread application of nanotechnology in agriculture remains challenging. Ensuring the scalability and cost-effectiveness of nanomanufacturing techniques remains a critical challenge to the large-scale adoption of nanotechnology in field applications. Moreover, environmental and safety concerns, such as the biodegradability of nanomaterials and the potential toxicity to soil microbiota and plants, must be thoroughly addressed to ensure sustainable and responsible use. Understanding the long-term impacts of nanomaterials on ecosystems is essential for minimizing risks and optimizing the benefits of applying nanotechnology to agricultural settings.

Future work should focus on specific areas such as improving the scalability of nanofabrication techniques, reducing the costs of nanomaterial production, and addressing environmental concerns through the development of biodegradable nanomaterials. Additionally, exploring the synergy between nanotechnology and AI, synthetic biology, and IoT technologies could lead to more efficient and sustainable precision farming practices. Further investigation into the long-term impacts of nanomaterials on ecosystems and their potential to improve crop resilience under climate change is also needed. The integration of these advanced technologies will enhance resource efficiency, improve crop health, and reduce the environmental footprint of agricultural practices. As research continues to evolve, nanotechnology will play an increasingly pivotal role in ensuring food security and sustainability, particularly in the face of global challenges such as climate change and population growth.

## Figures and Tables

**Table 1:** Overview of nanofabrication and microfluidics applied in plant-microbe research.

Technology	Description	Application in Agriculture	Advantages	Limitations	References
Soft lithography	Involves the formation of a mold using an elastomeric material (e.g., PDMS) to transfer micro- and nanoscale patterns onto substrates such as silicon or plastic	Used to fabricate microfluidic devices and nanopatterned surfaces that influence microbial behavior. Natural root surfaces are mimicked to promote microbial colonization and modulate microbial communication, enhancing plant growth and stress resistance	Low cost, flexible, suitable for application to various substrate materials, and ideal for applications in biological systems	Limited resolution with respect to other methods; may not be suitable for certain high-precision applications	[23,33,34]
Nanoimprinting	A high-throughput method for transferring nanoscale patterns onto substrates by pressing a mold onto the material	Used to fabricate nanostructured surfaces for seed coatings, microbial inoculants, and drug delivery systems, improving plant–microbe interactions and agricultural productivity	High resolution, low cost, scalable, and applicable to various substrates	Requires high precision; molds can be expensive and slow in certain cases	[35,36]
Microfluidics	Involves the manipulation of small fluid volumes within micro-sized channels, generating controlled environments for biological studies	Essential for simulating the rhizosphere, microfluidic systems are used for high-throughput screening of plant–microbe interactions or for monitoring exudate release from plant roots	Enables the real-time observation of microbial behavior; efficient and scalable for plant–microbe studies	Requires a complex setup, can be expensive, and has high operational requirements to ensure precision	[37,38]

## Data Availability

Not applicable.
